# Steady state engineering of a two-level system by the mixed-state inverse engineering scheme

**DOI:** 10.1038/s41598-024-53726-5

**Published:** 2024-02-10

**Authors:** M. Z. Wang, W. Ma, S. L. Wu

**Affiliations:** School of Physics and Materials Engineering, Dalian Nationalities University, Dalian, 116600 China

**Keywords:** Quantum information, Quantum mechanics, Theoretical physics

## Abstract

The mixed-state inverse engineering scheme is a control scheme used for engineering the quantum state of a driven open quantum system from an initial steady state to a final steady state. In this paper, we present an analytical study of this scheme applied to the driven two-level model coupled to a heat reservoir. Typically, when the purity of the quantum state varies, incoherent control techniques are required for mixed-state engineering. However, we show that for both Markovian and non-Markovian dynamics, coherent control protocols can transfer the quantum state into the target state. This simplification comes at a cost, as the evolution of the quantum state must be limited to restricted conditions, resulting in special trajectories in its Hilbert space that connect the initial and target states.

## Introduction

Precisely controlling quantum systems, particularly open quantum systems, is a challenging and important concern in the fields of quantum science and technology, including quantum information^1,2^, quantum thermodynamics^3^, and quantum optics^4^. Due to their dissipative nature, one method for controlling open quantum systems is to construct a nontrivial steady state so that the quantum state will relax towards this state regardless of the initial states^5^. This method, known as dissipative preparation^5,6^ or quantum quench^7^, is widely used for engineering quantum states^8^ and understanding phases of matter and transitions between them^9^. However, in some cases, the relaxation to the steady state (the target state) is very slow due to the metastable state^10^, making the dissipative preparation impractical or even detrimental.

To overcome this issue, optimal unitary transformations can be performed on open quantum systems before they experience the relaxation process to remove the “population” on the lowest decaying dynamical mode in the initial state^11^. This results in an exponential acceleration of the relaxation of Markovian open quantum systems, similar to the Mpemba effect^12^. Alternatively, active driving via stochastic reset on the open quantum systems can also induce this exponential acceleration of the relaxation processs^13^. Furthermore, active control protocols, including coherent and incoherent control techniques^14,15^, can be used to drive the quantum state into the target steady state. The Shortcuts to adiabaticity for closed quantum systems provides a promised control scheme to accelerate the adiabatic evolution^16–19^, and has received extensive attention in recent years. The same idea has been generalized to the case of open quantum systems^14,20^. Shortcuts to adiabaticity (STAs) of open quantum systems are an advisable choice for steady state engineering^21^. For the steady state engineering, a special scheme known as the mixed-state engineering scheme is directly related^14,22^. However, it is troublesome that the control protocol given by the mixed-state engineering is always unavailable in the real-experimental setting, such as infinite pulse strengths, negative decay rates^23,24^, even unrealistic couplings to the heat reservoir^22^.

In this paper, we apply the mixed-state inverse engineering scheme to open two-level systems to demonstrate that practicable control protocols can be obtained by designing proper trajectories from the initial state to the target state. For Markovian dynamics, a classical coherent field combined with a smoothly tunable environment temperature can drive the two-level systems along the adiabatic trajectory given by the instantaneous steady state of the reference Liouvillian. If the environment temperature is fixed as a constant, we show that a coherent control protocol is sufficient to transfer the quantum state into the target steady state, but the quantum state trajectory must satisfy particular constraint conditions. A similar result is obtained for non-Markovian dynamics. Without specifying the time-dependent decay rate and the Lamb shift, the coherent control protocol successfully transfers the quantum state from the initial “steady state” to the final one. Here, we call the eigenstate of the Liouvillian superoperator with zero eigenvalue as the instantaneous steady state of the non-Markovian quantum system.

The remainder of this paper is organized as follows. In Section 2, we briefly review STAs of open quantum systems and the mixed-state inverse engineering scheme. We apply the mixed-state inverse engineering scheme to two-level systems for Markovian dynamics and present a control protocol with a coherent control pulse and a tunable environmental temperature as incoherent control in Section 3. Coherent control protocols are presented in Section 4, where we show that different choices on the quantum state trajectory always accompany different constraint conditions. In Section 5, we present coherent control protocols for two-level non-Markovian systems, illustrating that open quantum systems with non-Markovian dynamics can still be exactly controllable. We conclude this paper in Section 6.

## The steady state inverse engineering

In this section, we briefly review the mixed-state inverse engineering scheme^22^, and apply it in the steady state engineering.

Let us consider an open quantum system whose dynamics is governed by the following time-convolutionless master equation in the superoperator description,

1 $$\begin{aligned} \partial _{t}|\rho (t)\rangle \rangle =\hat{{\mathcal {L}}_{c}}(t)|\rho (t)\rangle \rangle , \end{aligned}$$

where $\hat{{\mathcal {L}}}_c (t)$ is a time-dependent control Liouvillian superoperator and $|\rho (t)\rangle \rangle$ is the density matrix vector. A dynamical invariant $\hat{{\mathcal {I}}} (t)$ of such open quantum system is defined as a superoperator which satisfies^25^

2 $$\begin{aligned} \partial _t \hat{{\mathcal {I}}}(t)-[\hat{{\mathcal {L}}_{c}}(t),\hat{{\mathcal {I}}}(t)]=0. \end{aligned}$$

$\hat{{\mathcal {I}}} (t)$ can be formulated in Jordan canonical form with biorthogonal eigenvectors which satisfies^20,26^

$$\begin{aligned} \hat{{\mathcal {I}}}\,|D_\alpha ^{(i)}\rangle \rangle= & {} \lambda _\alpha |D_\alpha ^{(i)} \rangle \rangle +|D_\alpha ^{(i-1)}\rangle \rangle ,\\ \langle \langle E_\alpha ^{(i)}| \hat{{\mathcal {I}}}= & {} \lambda _\alpha \langle \langle E_\alpha ^{(i)}|+\langle \langle E_\alpha ^{(i+1)}|, \end{aligned}$$

with $i=0,1,...,n_\alpha -1$. $|D_{\alpha }^{(i)}(t)\rangle \rangle$ and $\langle \langle E_{\alpha }^{(i)}(t)|$ are the right and left eigenvectors of $\hat{{\mathcal {I}}}(t)$ in the Hilbert-Schmidt space with the eigenvalue $\lambda _{\alpha }$. We also assume that the $\alpha$-th Jordan block is $n_\alpha$-dimensional. Generally, $\hat{{\mathcal {I}}}(t)$ can be used to generate an arbitrary solution of the time-convolutionless master equation. The general solution of Eq. (1) can be written as^14^

3 $$\begin{aligned} |\rho (t)\rangle \rangle =\sum ^{m-1}_{\alpha =0}a_{\alpha }e^{\eta _{\alpha }(t)}|\Phi _{\alpha }(t)\rangle \rangle . \end{aligned}$$

$|\Phi _{\alpha }(t) \rangle \rangle$ is a right vector in the $\alpha$th Jordan block, which can be written as

4 $$\begin{aligned} |\Phi _{\alpha }(t)\rangle \rangle =\sum ^{n_{\alpha }-1}_{i=0}b_{i}^{\alpha }(t)|D_{\alpha }^{(i)}(t)\rangle \rangle . \end{aligned}$$

$\eta _{\alpha }(t)$ is a complex phase defined as

5 $$\begin{aligned} \eta _{\alpha }(t)=\int _0^t\langle \langle \Psi _\alpha (t')|\left( \hat{{\mathcal {L}}_{c}}(t')-\partial _{t'}\right) |\Phi _\alpha (t')\rangle \rangle dt', \end{aligned}$$

where $\langle \langle \Psi _{\alpha }(t)|$ is a left vector of the $\alpha$-th Jordan block, which satisfies $\langle \langle \Psi _{\beta }(t)| \Phi _{\alpha }(t)\rangle \rangle =\delta _{\alpha \beta }$. $a_{\alpha }$ is time-independent coefficient, which is given by $a_{\alpha }= \langle \langle \Psi _{\alpha }(0)|\rho (0)\rangle \rangle$.

As a reference, we consider an adiabatic evolution of a quantum system governed by the reference Liouvillian $\hat{{\mathcal {L}}}_{0}(t)$. Here, we do not limit the dynamics governed by $\hat{{\mathcal {L}}}_{0}(t)$ to be Markovian, but the corresponding evolution must be a completely positive trace-preserving map. Furthermore, we assume that $\hat{{\mathcal {L}}}_{0}(t)$ admits a unique (instantaneous) steady state $\rho _{0}(t)$, which satisfies

$$\begin{aligned} \hat{{\mathcal {L}}}_{0}(t)|\rho _{0}(t)\rangle \rangle =0. \end{aligned}$$

When the adiabatic evolution condition is satisfied, the quantum state can be transferred form the initial steady state to the final steady state with a satisfactory fidelity. To accelerate the adiabatic steady state process, we consider that the invariant has only one 1-dimensional Jordan block with nonzero eigenvalue $\lambda _{s}$, i.e., $\hat{{\mathcal {I}}}(t)=\lambda _{s}|\Phi _{0}(t)\rangle \rangle \langle \langle I|,$ where $\lambda _s$, where $\langle \langle I|$ is the left vector corresponding to an identity matrix. If we set $|\Phi _{0}(0)\rangle \rangle =|\rho _{0}(0)\rangle \rangle$ and $|\Phi _{0}(t_{f})\rangle \rangle =|\rho _{0}(t_{f})\rangle \rangle$, the eigenvector $|\Phi _{0}(t)\rangle \rangle$ corresponds to trajectory connecting the initial steady state and the target steady state. Since $|\Phi _{0}(t)\rangle \rangle$ should be a quantum state of the open quantum system, we can expand $|\Phi _{0}(t)\rangle \rangle$ by SU(*N*) Hermitian generators $\{|T_{\mu }\rangle \rangle \}^{N^{2}-1}_{\mu =1}$, i.e.,

6 $$\begin{aligned} |\Phi _{0}(t)\rangle \rangle =\frac{1}{N}\left( |I\rangle \rangle +\sqrt{\frac{N(N-1)}{2}} \sum _{\mu =1}^{N^2-1}r_{\mu }|T_\mu \rangle \rangle \right) , \end{aligned}$$

where $\textbf{r}=(r_{1}, r_{2},..., r_{N^{2}-1})$ is the generalized Bloch vector, and $|I\rangle \rangle$ is the right vector corresponding to an $N \times N$ identical operator.

To formulate feasible control protocols, we impose that the control Liouvillians take the form as

7 $$\begin{aligned} \hat{ {\mathcal {L}}}_c[\bullet ]=-i[{H}(t),{\bullet }]+\sum _\alpha \hat{{\mathcal {D}}}[L_\alpha ](\bullet ), \end{aligned}$$

in which *H*(*t*) is the control Hamiltonian, and

8 $$\begin{aligned} \hat{{\mathcal {D}}}[L_{\alpha }](\rho )=L_{\alpha }(N_{\alpha })\rho L_{\alpha }^{\dag }(N_{\alpha }) -\frac{1}{2}\{L_{\alpha }^{\dag }(N_{\alpha }) L_{\alpha }(N_{\alpha }),\rho \} \end{aligned}$$

is the control Lindbladian. The Lindblad operators $L_{\alpha }(N_{\alpha })$ are related to some incoherent control parameters $N_{\alpha }$, such as the decoherence rates and the temperatures of the environments, which will be used in the later discussion on the control Liouvillian. Since the Hamiltonian $H_c(t)$ can be expanded in terms of SU (N) Hermitian generators $\{T_k\}$, i.e.,

$$\begin{aligned} H_c (t) =\sum _{k=1}^{N^2-1}c_k(t)T_k, \end{aligned}$$

we might choose $\{c_k (t)\}$ as the coherent control parameters. In this way, the control Liouvillians can always present feastible control protocols. Substituting $\hat{{\mathcal {L}}}_c(t)$ and $\hat{{\mathcal {I}}} (t)$ into Eq. (2), we can express the control parameters $\{c_k, N_\alpha \}$ as a function of the generalized Bloch vector and its derivative $\{r_\mu , \partial _t r_\mu \}$. On the other hand, we impose that the control Liouvillian is always the same as the reference Liouvillian at the initial and final moment, which leads to boundary conditions for $\{r_\mu ,\partial _t r_\mu \}$. By utilizing the control parameters $\{c_k, N_\alpha \}$ and setting proper boundary conditions for $\{r_\mu , \partial _t r_\mu \}$, the open quantum system can be transferred from the initial steady state into the target steady state along an exact trajectory given by $|\Phi _0 (t) \rangle \rangle$. Here, we do not require $\left[ \hat{ {\mathcal {L}}}_c(0),\hat{ {\mathcal {I}}}(0)\left]=\right[\hat{ {\mathcal {L}}}_c(t_f),\hat{ {\mathcal {I}}}(t_f)\right] =0$, because of the difficulties in engineering parameters in the Lindbladian superoperator.

## The mixed-state inverse engineering scheme with the incoherent control

Consider a two-level system with a reference Hamiltonian $H_0(t)=\frac{ \omega _0}{2}\sigma _z+H_c(t),$ where $\omega _0$ is frequency difference, $H_c(t)$ is general control Hamiltonian, and $\sigma _z$ is *z* component of the Pauli matrix. The two-level system couples to a bosonic heat reservoir at finite temperature $T_0$. In the interaction picture respecting to $\frac{ \omega _0}{2}\sigma _z$, the dynamics of the two-level system is governed by a following phenomenological Markovian master equation^28^,

9 $$\begin{aligned} \partial _t \rho (t)= & {} \hat{{\mathcal {L}}}_{0}(t)[\rho (t)]\nonumber \\= & {} -i[H_{c0}^I(t),\rho (t)]+\gamma (N_0+1)\hat{{\mathcal {D}}}[\sigma _-](\rho (t))\nonumber \\{} & {} +\gamma N_0 \hat{ {\mathcal {D}}}[\sigma _+](\rho (t)), \end{aligned}$$

in which $\gamma$ denotes the decoherence strength , and $N_0=[\exp (\omega _0/ T_0)-1]^{-1}$ is the mean excitation number. Throughout this paper, we assume the dimensionless units $\hbar =k_B=1$. The reference Hamiltonian is expressed as $H_{c0}^I(t)=\Omega _0(t)\sigma _x$ with the time-dependent control field strength $\Omega _0(t)$.

Here, we use the ”bra-ket” notation for superoperator^29^. On the one hand, the density matrix can be reshaped into $1\times 4$ density matrix vector. The density matrix vector can be written as $|\rho (t)\rangle \rangle =(\rho _{00}(t),\rho _{01}(t),\rho _{10}(t), \rho _{11}(t))^{{\textbf {T}}}$, where $\rho _{ij}(t)= \langle i|\rho (t)|j\rangle$ with $i=0,\,1$. $|0\rangle$ and $|1\rangle$ are the eigenstates of $\sigma _z$. On the other hand, the reference Liouvillian superoperator $\hat{{\mathcal {L}}}_0(t)$ (Eq. (9)) can be expressed as a $4\times 4$ matrix,

10 $$\begin{aligned} \hat{{\mathcal {L}}}_0(t)=\gamma \left( \begin{array}{cccc} -N_0-1 &{}\frac{i\Omega _0}{2\gamma } &{} -\frac{i\Omega _0}{2\gamma }&{}N_0 \\ \frac{i\Omega _0}{2\gamma } &{} -\frac{2N_0+1}{2}&{}0 &{} -\frac{i\Omega _0}{2\gamma }\\ -\frac{i\Omega _0}{2\gamma } &{}0 &{}-\frac{2N_0+1}{2} &{}\frac{i\Omega _0}{2\gamma } \\ N_0+1 &{} -\frac{i\Omega _0}{2\gamma }&{} \frac{i\Omega _0}{2\gamma }&{}-N_0 \\ \end{array} \right) . \end{aligned}$$

The steady state of two-level system is given by the condition $\hat{{\mathcal {L}}}_0(t)|\rho _0(t)\rangle \rangle =0$, which results in

11 $$\begin{aligned} |\rho _0\rangle \rangle =\frac{1}{p}\left( \begin{array}{cccc} N_0(2N_0+1)+(\Omega _0/\gamma )^2 \\ -i \Omega _0/\gamma \\ i \Omega _0/\gamma \\ (N_0+1)(2N_0+1)+(\Omega _0/\gamma )^2 \\ \end{array} \right) , \end{aligned}$$

with the normalization factor $p=(2N_0+1)^2+2(\Omega _0/\gamma )^2$. Since the corresponding left eigenstate fulfills $\hat{{\mathcal {L}}}_0^\dagger (t)|\rho _0'(t)\rangle \rangle =0$, one can obtain $\langle \langle \rho _0'|=\langle \langle I|=(1,0,0,1)$, which leads to $\langle \langle \rho _0'|\rho _0\rangle \rangle =1$.

In what follows, we parameterize the eigenvalue and eigenvector of the invariant $\hat{{\mathcal {I}}}(t)$. For a two-level system, the eigenvector can be parameterized as,

12 $$\begin{aligned} | {{\varrho }}(t)\rangle \rangle =\frac{1}{2}\left( | I \rangle \rangle +\sum _{i=x,y,z} r_i |\sigma _i \rangle \rangle \right) , \end{aligned}$$

where $r_i$ is the *i*-th component of the Bloch vector, and $\sigma _i$ is *i*-component of the Pauli operators. And we set $\lambda _s$ as an arbitrary constant with units of energy. Thus $\hat{{\mathcal {I}}}(t)$ reads

13 $$\begin{aligned} \hat{{\mathcal {I}}}(t)=\frac{\lambda _s}{2} \left( \begin{array}{cccc} 1+r_{z} &{} 0 &{} 0 &{} 1+r_{z}\\ r_{x}-i\,r_{y} &{} 0 &{} 0 &{} r_{x}-i\,r_{y}\\ r_{x}+i\,r_{y} &{} 0 &{} 0 &{} r_{x}+i\,r_{y}\\ 1+r_{z} &{} 0 &{} 0 &{} 1+r_{z} \end{array}\right) . \end{aligned}$$

Assume that the control Liouvillian $\hat{{\mathcal {L}}}_c(t)$ can be written as

14 $$\begin{aligned} \partial _t \rho (t)= & {} \hat{{\mathcal {L}}}_{c}(t)[\rho (t)]\nonumber \\= & {} -i[H_{c}^I(t),\rho (t)]+\gamma (N+1)\hat{{\mathcal {D}}}[\sigma _-](\rho (t))+\gamma N \hat{ {\mathcal {D}}}[\sigma _+](\rho (t)), \end{aligned}$$

with $H_c^I(t)=\Delta (t)\sigma _z+\Omega (t)\sigma _x$, and and $N=[\exp (\omega _0/ T)-1]^{-1}$. Here we also assume that the temperature *T* of the heat bosonic reservoir is tunable, which is the incoherent control parameter in Eq. (8). Following the standard procedure, the control parameters and the boundary conditions of the inverse engineering protocol can be identified. Substituting the dynamical invariant $\hat{{\mathcal {I}}}(t)$ and the control Liouvillian $\hat{{\mathcal {L}}}_c(t)$ into Eq. (1), we have

15a $$\begin{aligned} \Omega (t)&=\frac{\gamma \,\left( r_{x}^{2}+r_{y}^{2}\right) -{r_{z}}\,\partial _{t}\left( r_{x}^{2}+{r_{y}^{2}}\right) +\left( r_{x}^{2}+r_{y}^{2}\right) \,\partial _{t}r_{z}}{2\,{r_{y}} \,\left( {r}^{2}+r_{z}^{2}\right) }, \end{aligned}$$

15b $$\begin{aligned} \Delta (t)&=\frac{\left( {r_{x}}\left( {r_{y}}{\partial _{t}r_{y}}+{r_{z}}\partial _{t}{r_{z}}\right) -\partial _{t}{r_{x}} \left( r_{y}^{2}+2 r_{z}^{2}\right) +\gamma {r_{x}}{r_{z}}\right) }{2\,{r_{y}}\,\left( {r}^{2}+{r_{z}}^{2}\right) }, \end{aligned}$$

15c $$\begin{aligned} N(t)&=-\left( \frac{1}{2}+\frac{{r_{z}}}{{r}^{2}+{r_{z}}^{2}}+\frac{\partial _{t}r^{2}}{2\gamma \, \left( {r}^{2}+{r_{z}}^{2}\right) }\right) , \end{aligned}$$

with $$r=\sqrt{r_x^2+r_y^2+r_z^2}$$.

Since our purpose is to accelerate the adiabatic steady state process governed by $\hat{{\mathcal {L}}}_0(t)$, we only require that the initial state and the target state are same for the adiabatic engineering and the inverse engineering, but the trajectory from the initial state and the target state can be chosen freely. To make $|\rho _0(t)\rangle \rangle$ and $| \mathcal \varrho (t)\rangle \rangle$ equal at the beginning and the end of the control process, we impose the initial boundary conditions

$$\begin{aligned} r_x(0)= & {} 0,\\ r_y(0)= & {} \frac{2\Omega _0(0)/\gamma }{(2N_0+1)^2+2\Omega _0(0)^2/\gamma ^2},\\ r_z(0)= & {} -\frac{2N_0+1}{(2N_0+1)^2+2\Omega _0(0)^2/\gamma ^2}, \end{aligned}$$

and the final boundary conditions

$$\begin{aligned} r_x(t_f)= & {} 0,\\ r_y(t_f)= & {} \frac{2\Omega _0(t_f)/\gamma }{(2N_0+1)^2+2\Omega _0(t_f)^2/\gamma ^2},\\ r_z(t_f)= & {} -\frac{2N_0+1}{(2N_0+1)^2+2\Omega _0(t_f)^2/\gamma ^2}. \end{aligned}$$

Substituting the above boundary conditions into Eq. (15), it yields

16 $$\begin{aligned} \partial _t r_i(0)=0,\, \partial _t r_i(t_f)=0,\,(i=x,y,z). \end{aligned}$$

The trajectory of the quantum state is determined by parameterizing the Bloch vector, which can be designed according to control tasks. For the adiabatic trajectory given by the instantaneous steady state Eq. (11), the components of the Bloch vector read

17 $$\begin{aligned} r_x(t)= & {} 0,\nonumber \\ r_y(t)= & {} \frac{2\,\Omega _0(t)/\gamma }{\left( 2\,N_0+1\right) ^{2}+2\,{{\Omega _0(t)^{2}/\gamma ^{2}}}},\nonumber \\ r_z(t)= & {} -\frac{2N_0+1}{(2N_0+1)^2+2\Omega _0(t)^2/\gamma ^2}. \end{aligned}$$

We select the following profile for $\Omega _0(t)$,

$$\begin{aligned} \Omega _0(t)=\Omega _c \left( 1-6\,\frac{t^2}{t_f^2}\left( \frac{1}{2}-\frac{t}{3t_f}\right) \right) , \end{aligned}$$

where $\Omega _c$ is a constant with units of energy. This profile is corresponding to turning down the reference control field from $\Omega _c$ to 0 smoothly. Since $\partial _t\Omega _0(t)|_{t=t_0}=0$ and $\partial _t\Omega _0(t)|_{t=t_f}=0$, the boundary conditions Eq. (16) are satisfied. Taking Eq. (17) into Eq. (15), we can obtain the control parameters in analytic expression.

As mentioned in the adiabatic theorem, when the control field $\Omega _0(t)$ is varied slowly enough, the quantum state of the two-level system will track the trajectory of the instantaneous steady state into the target state^27^. Here we denote this trajectory as *“an adiabatic trajectory”*. Since $r_x(t)=0$ and $\partial _t r_x(t)=0$, the detuning $\Delta (t)$ absents for the adiabatic trajectory tracking. Therefore, if we select the adiabatic trajectory, the structure of the control Liouvillian $\hat{{\mathcal {L}}}_c(t)$ is completely the same as the reference Liouvillian $\hat{{\mathcal {L}}}_0(t)$.

**Figure 1:**
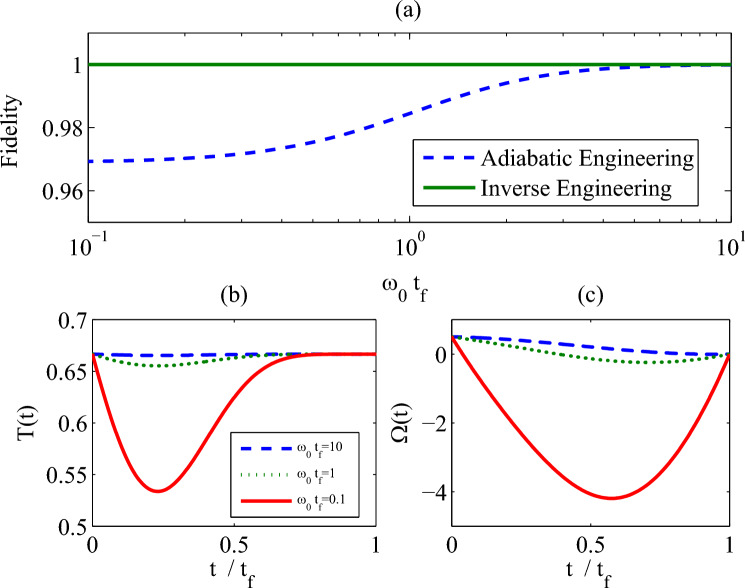
(**a**)The final infidelity vs $\omega _0 t_f$; (**b**) temperature of the reservoir *T*(*t*) and (**c**) the control field $\Omega (t)$ with time $t/t_f$. Parameters: $\Omega _c=0.5\omega _0$, $\gamma =\omega _0$. We set $\omega _0=1$ as the unit of the other parameters.

The fidelities with respect to the instantaneous steady state for both protocols are plotted in Fig. 1a. The numeral results illustrate that the inverse engineering protocol forbids the transition from the steady state $\rho _0(t)$ and the other part of the Hilbert-Schmidt space. When the adiabatic engineering protocol is considered, the population on the target steady state only occurs in the adiabatic limit. On the other hand, the control parameters in the control Liouvillian $\hat{{\mathcal {L}}}(t)$, i.e., the temperature of the bosonic heat reservoir *T*(*t*) and the control field $\Omega (t)$ are plotted in Fig.1b,c. For $t_f=10\omega _0^{-1}$, the control parameters are very close to the adiabatic case (see blue dash lines in Fig. 1b,c). With the decrease of $t_f$, the control parameters deviate from the adiabatic engineering protocol’s, and the target state can still be reached for arbitrary $t_f$ ( green solid line1 in Fig. 1a).

## Coherent control protocols

### The coherent control protocol via $r_x(t)$

In this section, we show that coherent control protocols is enough to drive the open quantum system into the target steady state. What we should do is to set *N*(*t*) as a constant $N_0=[\exp (\omega _0/ T_0)-1]^{-1}$. Thus the incoherent control absents, and there is only a coherent control field with the strength $\Omega (t)$ and the detuning $\Delta (t)$ in the control protocol.

Here, we select a designed $r_x$ to satisfy the constraint condition $N(t)=N_0$. According to Eq. (15c), $r_x$ has to satisfy the following differential equation,

18 $$\begin{aligned} \partial _{t}r_{x}^{2}+\left( 2\!N_0+1\right) \!\gamma \,r_{x}^{2}=-\Lambda (t), \end{aligned}$$

with

$$\begin{aligned} \Lambda (t)= & {} \left( 2\!N_0+1\right) \!\gamma \,r_{y}^{2}+2\,\gamma \,{r_{z}}\left( \left( 2N_0+1\right) \,{r_{z}}+1\right) \\{} & {} +\partial _{t}\left( r_{y}^{2}+r_{z}^{2}\right) . \end{aligned}$$

We may consider Eq. (18) as a differential equation about $r_x^2$. Solving this equation is equivalent to select a trajectory with the particular x-component of the Bloch vector. For formally solving the differential equation about $r_x$, we introduce a new variant ${\tilde{r}}_{x}=r_{x} e^{\left( 2\!N_0+1\right) \!\gamma \,t/2},$ which satisfies

19 $$\begin{aligned} \partial _{t} {{\tilde{r}}}_{x}^{2}=-\Lambda (t)e^{\left( 2\!N_0+1\right) \!\gamma \,t}. \end{aligned}$$

At last, we obtain the formal solution about $r_x^2$,

20 $$\begin{aligned} r_x^2=-\int _0^t d\tau \,\Lambda (\tau )e^{-\left( 2\!N_0+1\right) \!\gamma \,(t-\tau )}. \end{aligned}$$

Since $r_x$ is one component of the Bloch vector, the following restrictions have to be satisfied: (i) as $r_x$ is always real, $\int _0^t d\tau \,\Lambda (\tau )e^{\left( -2\!N_0+1\right) \!\gamma \,(t-\tau )}\le 0$ has to be ensured; (ii) $0\le r_x^2+r_y^2+r_z^2\le 1$. Those restrictions illustrate that the control parameters in $\hat{{\mathcal {L}}}_c(t)$ must be chosen carefully to make sure that the Bloch vector corresponds to a reasonable quantum state^30^.

By introducing two new variants, ${\tilde{r}}_{y}=r_{y}e^{\left( 2\!N_0+1\right) \!\gamma \,t/2},\ {\tilde{r}}_{z}=r_{z} e^{\left( 2\!N_0+1\right) \!\gamma \,t}$, we can transform Eq. (18) into the following form,

$$\begin{aligned} \partial _{t}{\tilde{r}}_{x}^{2}=-\left( \partial _{t}{\tilde{r}}{}_{y}^{2}+\partial _{t} {\tilde{r}}_{z}^{2}e^{-\left( 2\!N_0+1\right) \!\gamma \,t}+2\!\gamma \, {{\tilde{r}}_{z}}\right) \end{aligned}$$

Integrating above equation with time, it yields

$$\begin{aligned} {\tilde{r}}_{x}^{2}(t)-{\tilde{r}}_{x}^{2}(0)= & {} -{\tilde{r}}{}_{y}^{2}(t)+{\tilde{r}}{}_{y}^{2}(0)-2\, \gamma \,\int _{0}^{t}{{\tilde{r}}_{z}(\tau )}\,\text {d}\tau \\{} & {} -\int _{0}^{t}\partial _{t}{\tilde{r}}_{z}^{2}(\tau )e^{-\left( 2\!N+1\right) \!\gamma \,\tau }\,\text {d}\tau . \end{aligned}$$

By partial integration of the last term in above equation, one finds

$$\begin{aligned}{} & {} {\tilde{r}}_{x}^{2}(t)+{\tilde{r}}_{y}^{2}(t)+{\tilde{r}}_{z}^{2}(t)\,e^{-\left( 2\!N_0+1\right) \!\gamma \,t}\\{} & {} \quad ={\tilde{r}}_{x}^{2}(0)+{\tilde{r}}_{y}^{2}(0)+{\tilde{r}}_{z}^{2}(0)-\Theta (t), \end{aligned}$$

with

$$\begin{aligned} \Theta (t)=\!\!\!\int _{0}^{t}\!\!\!\!\left( \left( 2\!N_0+1\right) \gamma \,\!{\tilde{r}}_{z}^{2}(\tau )\, e^{-\left( 2N_0+1\right) \!\gamma \!\,\tau } +2\gamma \,{{\tilde{r}}_{z}(\tau )}\right) \,\!\!\text {d}\tau . \end{aligned}$$

Turning back into the Bloch vector, we obtain the relation

21 $$\begin{aligned} r^{2}(t)=\left( r^{2}(0)-\Theta (t)\right) \,e^{-\left( 2\!N_0+1\right) \!\gamma \,t}, \end{aligned}$$

with $$r(t)=\sqrt{r_x^2(t)+r_y^2(t)+r_z^2(t)}$$.

Now let us consider the nonadiabatic control protocol from the initial state $|\rho _0(t_f)\rangle \rangle$ to the target state $|\rho _0(t_f)\rangle \rangle$. We set the ansatz

22 $$\begin{aligned} r_i(t)=r_i(0)-6 (r_i(0)-r_i(t_f))\frac{t^2}{t_f^2}\left( \frac{1}{2}-\frac{t}{3t_f}\right) \end{aligned}$$

for $i=y,\,z$. And, $r_x(t)$ can be obtained by the exact solution Eq. (20), or by solving the differential equation Eq. (18) numerically. It is worth to notice that under such parameterized protocol the boundary conditions for *y* and *z* components of the bloch vector are satisfied automatically, but the boundary condition of $r_x(t_f)$ is not. As shown in Eq. (21), if $r_x(t_f)=0$, it requires

$$\begin{aligned} \left( r^{2}(0)-\Theta (t_f)\right) \,e^{-\left( 2\!N_0+1\right) \!\gamma \,t_f}-r_y^2(t_f)-r_z^2(t_f)=0. \end{aligned}$$

This means that $t_f$ can not be chosen arbitrarily. The final *x*-component of the bloch vector $r_x(t_f)$ is related to the decoherence strength $\gamma$ and the *z* component of the bloch vector $r_z(t)$. On the one hand, we can get control period $t_f$ numerically by optimization methods, such as gradient, conjugate direction and quasi-Newton methods^31^. On the other hand, since the trajectory of the quantum state can be chosen optionally, one may select a proper components $r_y(t)$ and $r_z(t)$ to ensure the final boundary condition^32^.

**Figure 2:**
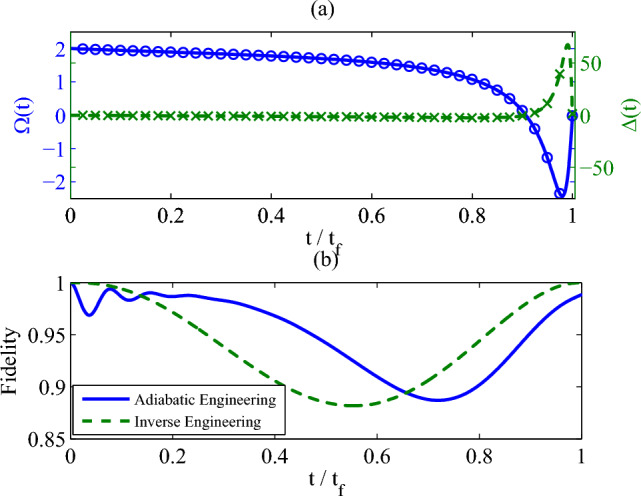
(**a**) The control field strength $\Omega (t)$ and the detuning $\Delta (t)$, (**b**) the fidelity vs the non-dimensional time $t/t_f$. Parameters: $\Omega (0)=2\omega _0$, $\gamma =2\omega _0$, $T_0=0$ and $t_f=10/\omega _0$. We set $\omega _0=1$ as the unit of the other parameters.

As an example, we select the parameters as follows: the strength of the initial control field $\Omega _c=2\omega _0$, the decoherence strength $\gamma =2\omega _0$, the temperature of the reservoir $T_0=0$, and $t_f=10\omega _0^{-1}$. The control field $\Omega (t)$ and the detuning $\Delta (t)$ are presented in Fig. 2a. As prediction, the control field and the detuning are turned off at the end of the control process. We also present the fidelity with respect to a reference state $\rho _r(t)=\frac{1}{2}\left( I+r_y(t)\sigma _y +r_z(t)\sigma _z\right) ,$ where $r_y(t)$ and $r_z(t)$ are given by Eq. (22). As shown in Fig. 2b (the green dash line), the quantum state deviate the trajectory of the reference state at beginning, which is due to the participation of $r_x(t)$ in the dynamical evolution; two trajectories converge to the target state at the final moment of time, which attributes to $r_x(t_f)=0$ at the end of the control process. Comparing with the adiabatic engineering protocol (the blue solid line), the final fidelity for the inverse engineering protocol with respect to the target steady state for the inverse engineering is perfect, far better than the adiabatic one.

### The coherent control protocol via $r_y(t)$

In this part, we show how to drive the quantum state into the target state by a coherent control protocol without the detuning $\Delta (t)$. As shown in Eq. (15), when $r_x(t)=0$ and $\partial _t r_x(t)=0$, the detuning $\Delta (t)$ equals to zero. Therefore, we consider a modification on $r_y(t)$. As required for the coherent control protocol, the main excitation number of the heat reservoir is constant, i.e., $N(t)=N_0$. And we set that $r_x(t)=0$ and $\partial _t r_x(t)=0$ at $\forall \,t$. The differential equation about $r_y^2(t)$ can be obtained by considering Eq. (15c)

23 $$\begin{aligned} \partial _{t}r_{y}^{2}+\left( 2\!N_0+1\right) \!\gamma \,{r_{y}}^{2}=-\Lambda '(t) \end{aligned}$$

with

24 $$\begin{aligned} \Lambda '(t)=2\,\gamma \,{r_{z}}\left( \left( 2N_0+1\right) \,{r_{z}}+1\right) +\partial _{t}r_{z}^{2}. \end{aligned}$$

At this time, the only control parameter used in the control Liouvillian $\hat{{\mathcal {L}}}_c(t)$ is the coherent control field $\Omega (t)$, which reads

25 $$\begin{aligned} \Omega (t)=\frac{\gamma \,r_{y}^{2}-{r_{z}}\,\partial _{t}{r_{y}^{2}}+r_{y}^{2}\,\partial _{t}r_{z}}{2\,{r_{y}} \,\left( {r_y}^{2}+2r_{z}^{2}\right) }. \end{aligned}$$

**Figure 3:**
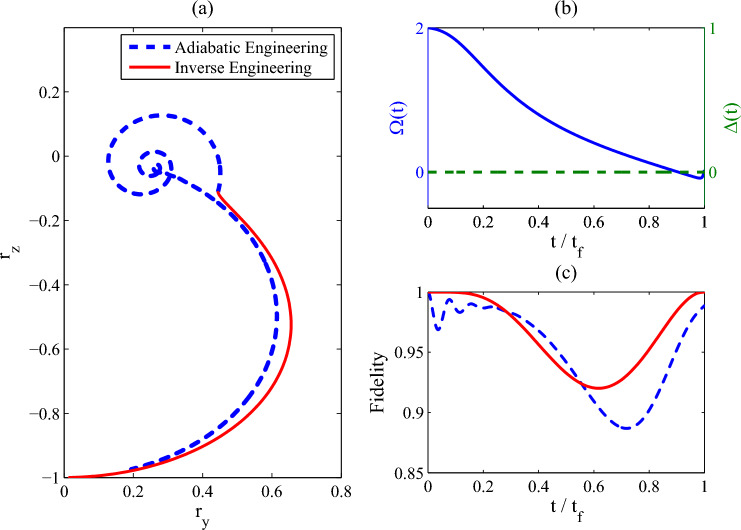
(**a**) the trajectory of the quantum state in the bloch sphere; (**b**) the control field strength $\Omega (t)$ and the detuning $\Delta (t)$, (**c**) the fidelity vs the non-dimensional time $t/t_f$. Parameters: $\Omega (0)=2\omega _0$, $\gamma =2\omega _0$, $T_0=0$ and $t_f=10\omega _0^{-1}$. We set $\omega _0=1$ as the unit of the other parameters.

The control task is still that the control field $\Omega _0(t)$ in the reference Liouvillian $\hat{{\mathcal {L}}}_0(t)$ decreases from $\Omega _c$ to 0. The numerical results are shown in Fig. 3, in which we present the trajectory of the quantum state in the Bloch sphere, the control parameters, and the fidelity between the quantum state $\rho (t)$ and the reference state $\rho _r(t)$. As shown in Fig. 3a, when the inverse mixed state engineering scheme is used (red solid line), the quantum system is driven into the target state at the end of the control process, which is also verified by the fidelity in Fig. 3c. In contrast, the quantum state engineered by the adiabatic engineering protocol can not reach the target state. What is more, the detuning for this protocol is always zero (see Fig. 3b), which means the control Liouvillian $\hat{{\mathcal {L}}}_c(t)$ has the same form with the reference Liouvillian $\hat{{\mathcal {L}}}_0(t)$ even for the coherent control protocol.

## The coherent control protocols for the non-markovian dynamics

### The control parameters

Now, we are in the position to study the non-Markovian case. Consider a two-level system with frequency $\omega _0$ driven by an external laser of frequency $\omega _L$. There is a detuning $\Delta =\omega _0-\omega _L$ between the two-level system and the external laser. The two-level atom is embedded in a bosonic reservoir at zero temperature. The reservoir has a Lorentzian spectral density, $J(\omega )=\frac{\gamma _0}{2\pi } \frac{\lambda ^2}{(\omega -\omega _0+\delta )^2+\lambda ^2},$ where $\delta =\omega _0-\omega _c$ is the detuning of $\omega _c$ to $\omega _0$, $\omega _c$ is the center frequency of the cavity, and $\lambda$ is the spectral width of the reservoir^28^. The parameter $\gamma _0$ is the decoherence strength of the system in the Markovian limit with a flat spectrum. The exact non-Markovian master equation can be written as^33^

26 $$\begin{aligned} \partial _t \rho (t)= & {} \hat{{\mathcal {L}}}_0(t)\rho (t)\nonumber \\= & {} -i[H_{0c}^I(t),\rho (t)]+\Gamma _0 (t) \hat{{\mathcal {D}}}[\sigma _-](\rho (t)), \end{aligned}$$

with the effective Hamiltonian $H_{0c}^I(t)=s_0(t)\sigma _+\sigma _-+\Omega _0(t)\sigma _x,$ where $s_0(t)$ and $\Omega _0(t)$ are the Lamb shift and the renormalized driving field respectively. The time-dependent decoherence strength $\Gamma _0(t)$ describes the dissipative non-Markovian dynamics due to the interaction between the system and reservoir. All these time-dependent coefficients can be given explicitly as follows

27 $$\begin{aligned} {s_0(t)=-\text {Im}\left[ \frac{\partial _t u(t)}{u(t)}\right] ,\! \Gamma _0(t)=-\text {Re}\left[ \frac{\partial _t u(t)}{u(t)}\right] }, \end{aligned}$$

with

28 $$\begin{aligned} {u(t)=k(t)\left[ \cosh \left( \frac{dt}{2}\right) +\frac{\lambda +i\delta }{d}\sinh \left( \frac{dt}{2}\right) \right] }, \end{aligned}$$

where $$k(t)=\exp (-(\lambda +2i\Delta -i\delta )t/2)$$ and $$d=\sqrt{(\lambda -i\delta )^2-2\gamma _0\lambda }$$.

If we use the “bra-ket” notation for superoperator, the reference Liouvillian superoperator $\hat{{\mathcal {L}}}_0(t)$ in Eq. (26) can be written as

29 $$\begin{aligned} \hat{{\mathcal {L}}}_0(t)=\Gamma _0\left( \begin{array}{cccc} -2&{} i\frac{{\Omega _{0}}}{\Gamma _{0} } &{} -i\frac{{\Omega _{0}}}{\Gamma _{0} }&{} 0\\ i\frac{{\Omega _{0}}}{\Gamma _{0}}&{} -1-i\frac{{s_{0}}}{\Gamma _{0}}&{} 0 &{} -i\frac{{\Omega _{0}}}{\Gamma _{0}}\\ -i\frac{{\Omega _{0}}}{\Gamma _{0} } &{} 0 &{} -1+i\frac{{s_{0}}}{\Gamma _{0} } &{} i\frac{{\Omega _{0}}}{\Gamma _{0}}\\ 2&{} -i\frac{{\Omega _{0}}}{\Gamma _{0} } &{} i\frac{{\Omega _{0}}}{\Gamma _{0} } &{} 0 \end{array}\right) . \end{aligned}$$

Also, the instantaneous steady state can be calculated by considering $\hat{{\mathcal {L}}}_0(t)|\rho _0\rangle \rangle =0$, which yields

30 $$\begin{aligned} |\rho _0\rangle \rangle =\frac{1}{z} \left( \begin{array}{c} {{{\Omega _0}}^2}{}\\ -{{\Omega _0}\, \left( {s_0 } + {i}\Gamma _0\right) }\\ -{{ \Omega _0}\, \left( {s_0 } - {i}\Gamma _0\right) }\\ \Gamma _0^2 + {{s_0 }}^2+ \, {{ \Omega _0}}^2 \end{array}\right) \end{aligned}$$

with the normalization factor $z=\Gamma _0^2 + {{s_0 }}^2+ 2\, {{\Omega _0}}^2$.

Here, we parametrize the dynamical invariants by the Bloch vectors as Eq. (12). Assume the control Liouvillian $\hat{{\mathcal {L}}}(t)$ with the same form as Eq. (26), i.e.,

31 $$\begin{aligned} \hat{{\mathcal {L}}}_c(t)\rho (t)=-i[H_{c}^I(t),\rho (t)]+\Gamma (t) \hat{{\mathcal {D}}}[\sigma _-](\rho (t)), \end{aligned}$$

with

32 $$\begin{aligned} H_c^I(t)=s(t)\sigma _+\sigma _-+\Omega (t)\sigma _x. \end{aligned}$$

The control parameters $\Gamma (t)$, *s*(*t*) and $\Omega (t)$ can be obtained by the standard procedure of the mixed state inverse engineering scheme,

33a $$\begin{aligned} \Omega (t)&=\frac{\left( {r_{x}}^{2}+{r_{y}}^{2}\right) \partial _{t}{r_{z}} -\left( \,\partial _{t}r_{x}^2+\partial _{t}r_{y}^2\right) \left( 1+r_{z}\right) }{2\, {r_{y}}\,\left( {r_{x}}^{2}+{r_{y}}^{2}+2\,{r_{z}}^{2}+2\,{{r_{z}}}\right) }, \end{aligned}$$

33b $$\begin{aligned} s(t)&=\frac{{r_{x}}\left( \partial _{t}r_{y}^{2}+\partial _{t}r_{z}^{2}\right) -2\partial _{t}r_{x}\left( {r_{y}}^{2}+2\,{r_{z}}^{2}+2\,r_{z}\right) \,}{2\,{r_{y}}\,\left( {r_{x}}^{2}+{r_{y}}^{2}+2\,{r_{z}}^{2}+2\,{{r_{z}}}\right) }, \end{aligned}$$

33c $$\begin{aligned} \Gamma (t)&=-\frac{\left( \partial _{t}r_{x}\,r_{x}\,+\partial _{t}r_{y}\,r_{y}\, +\partial _{t}r_{z}\,r_{z}\,\right) }{{r_{x}}^{2}+{r_{y}}^{2}+2\,{r_{z}}^{2} +2\,{{r_{z}}}}. \end{aligned}$$

### The coherent control protocol via $r_x$

Our purpose is to find a set of feasible control parameters to ensure that the quantum state can track the trajectory we designed. Here, we consider following trajectory. Firstly, the *y* and *z* components of the trajectory are set to be

34 $$\begin{aligned} r_y(t)= & {} -\frac{2\,\Omega _0(t)\Gamma _0(t)}{s_0(t)^2+2\Omega _0(t)^2+\Gamma _0(t)^2},\nonumber \\ r_z(t)= & {} \frac{\,s_0(t)^2+\Gamma _0(t)^2}{s_0(t)^2+2\Omega _0(t)^2+\Gamma _0(t)^2}, \end{aligned}$$

which are the *y* and *z* components of the Bloch vector given by the instantaneous steady state $|\rho _0(t)\rangle \rangle$. However, we can not select $r_x(t)$ as the *x*-component of the Bloch vector given by $|\rho _0(t)\rangle \rangle$ , due to ${r}^{2}+{r_{z}}^{2}+2\,{{r_{z}}} =0$ which results in meaninglessness control parameters (see Eq. (33)). Moreover, it is difficult to tune the time-dependent decoherence strength $\Gamma (t)$ properly in the experiment. Therefore, the decoherence strength can not be tuned artificially, i.e., $\Gamma (t)=\Gamma _0(t)$. Thus, according to Eq. (33c), it yields a differential equation about $r_x^2(t)$, which reads

35 $$\begin{aligned} \partial _{t}r_{x}^{2}+2\Gamma _{0}{r_{x}}^{2}=-\partial _{t}(r_{y}^{2}+r_{z}^{2}) -2\Gamma _{0}\left( {r_{y}}^{2}+2\,{r_{z}}^{2}+2\,{\mathrm {r_{z}}}\right) . \end{aligned}$$

Beside with the initial condition $r_x(0)=2\Delta \Omega _0(0)/(\Delta ^2+\Omega _0(0)^2)$, we can determine the *x*-component of the trajectory, i.e.,$r_x(t)$.

**Figure 4:**
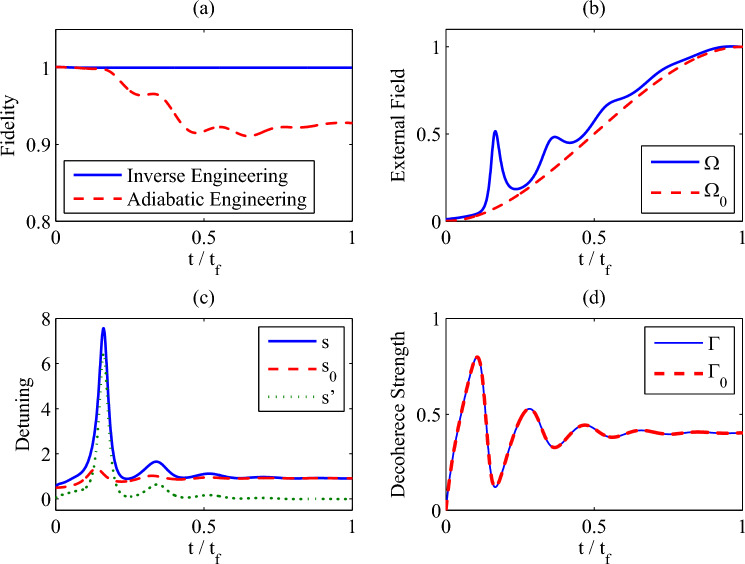
(**a**) The evolution of the fidelity of the inverse mixed state engineering protocol (the blue solid line) and the adiabatic engineering protocol (the red dash line), (**b**) the external control field strength, (**c**) the detuning, and (**d**) the decoherence strength) as a function of the dimensionless time $t/t_f$. Parameters: $\gamma _0=\omega _0,\,\lambda =0.5\omega _0,\,\Delta =0.5\omega _0, \,\delta =0.1\omega _0,\,\Omega _c=\omega _0, t_f=30/\omega _0$. We set $\omega _0=1$ as the unit of the other parameters.

Consider that the reference renormalized control field $\Omega _0(t)$ tunes up from 0 to a finite strength $\Omega _c$. The concrete profile of $\Omega _0(t)$ is chosen as ${\Omega _0(t)=6\Omega _c \,\frac{t^2}{t_f^2}\left( \frac{1}{2}-\frac{t}{3t_f}\right) .}$ The numeral results are presented in Fig. 4. As shown in Fig. 4a, for the inverse engineering scheme (the blue solid line), the quantum state of the open two-level system strictly follows the trajectory we designed. Instead, for the adiabatic engineering protocol (the red dash line), i.e., $\Gamma =\Gamma _0$, $\Omega =\Omega _0$, and $s=s_0$, there is severe fidelity loss. On the other side, the control field $\Omega (t)$ and the detuning *s*(*t*) deviate from $\Omega _0(t)$ and $s_0(t)$ respectively, but they coincide with each other at the beginning and the end (see Fig. 4b,c). This attributes to $\partial _t \Omega _0(0)=0$ and $\partial _t \Omega _0(t_f)=0$ . And $\Gamma (t)$ and *s*(*t*) become stable at the end, which are illustrated in Fig. 4c, d. If $t_f$ is not large enough, the control field $\Omega (t_f)$ and the detuning $s(t_f)$ can not coincide with $\Omega _0(t_f)$ and $s_0(t_f)$, but the fidelity never get loss. As we required, the spectral density can not be tuned, which requires $\Gamma (t)=\Gamma _0(t)$ all the time. Numerical results in Fig. 4d confirm this requirement, which shows that it is not necessary to engineer the reservoir for obtaining perfect fidelity. At last, *s*(*t*) contains two different contributions. One is the Lamb Shift $s_0(t)$, and the other comes form the detuning of the control field $s'(t)$. It is easy to obtain the detuning via $s'(t)=s(t)-s_0(t)$, which is illustrated by the green dot line in Fig.4c.

### The coherent control protocol via $r_y$

As shown in Eq. (33), the control parameters are related with $r_y^{-1}$, which cause singular points of the control parameter if $r_y=0$. This can be avoided by picking up a desired trajectory of $r_y$. Here, we consider that $r_x$ and $r_z$ are tracking the instantaneous steady state,

36 $$\begin{aligned} r_x(t)= & {} -\frac{2\,\Omega _0(t)s_0(t)}{s_0(t)^2+2\Omega _0(t)^2+\Gamma _0(t)^2},\nonumber \\ r_z(t)= & {} -\frac{\,s_0(t)^2+\Gamma _0(t)^2}{s_0(t)^2+2\Omega _0(t)^2+\Gamma _0(t)^2}. \end{aligned}$$

And $$r_y$$ satisfies

37 $$\begin{aligned} \partial _{t}r_{y}^{2}+2\Gamma _{0}{r_{y}}^{2}=-\partial _{t}(r_{x}^{2}+r_{z}^{2})-2\Gamma _{0}\left( {r_{x}}^{2}+2\,{r_{z}}^{2} +2\,{{r_{z}}}\right) , \end{aligned}$$

which corresponds to the constraint condition $\Gamma (t)=\Gamma _0(t)$.

**Figure 5:**
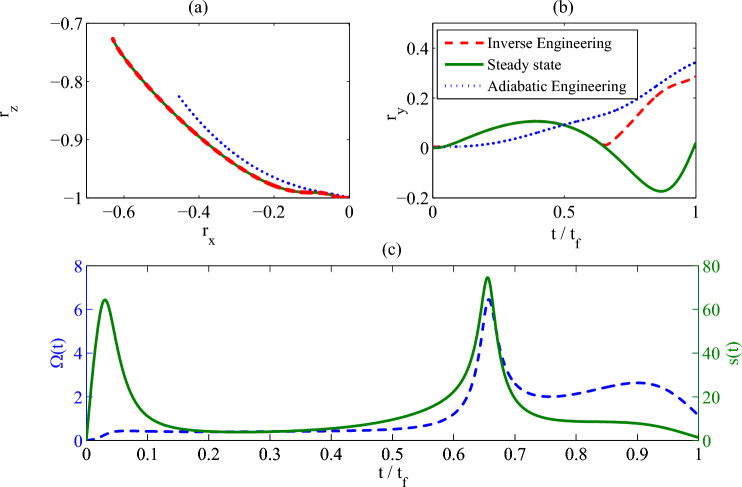
(**a**) The *x* and *z*-components and (**b**) *y*-component of the trajectory of both the instantaneous steady state given by Eq. (30) (the green solid line) and the quantum state engineered by the inverse engineering protocol (the read dash line) and the adiabatic engineering protocol (the blue dot line); (**c**) the external control field strength and the detuning as a function of the dimensionless time $t/t_f$. Parameters: $\gamma _0=10\omega _0,\,\lambda =0.2\omega _0,\,\Delta =0.375\omega _0,\,\delta =0.1\omega _0,\,\Omega _c=\omega _0, t_f=1.25/\omega _0$. We set $\omega _0=1$ as the unit of the other parameters.

The control task is to increase the control field $\Omega _0(t)$ in the reference Liouvillian $\hat{{\mathcal {L}}}_0(t)$ from 0 to $\Omega _c$. The profile of $\Omega _0(t)$ is chosen as ${\Omega _0(t)=6\Omega _c \,\frac{t^2}{t_f^2}\left( \frac{1}{2}-\frac{t}{3t_f}\right) .}$ The parameters for the non-Markovian dynamics are assumed to be $t_f=1.25/\omega _0$, $\Omega _c=\omega _0$, $\gamma _0=10\omega _0$, $\Delta = 0.375\gamma _0$, $\lambda =0.2\omega _0$, and $\delta =0.1\gamma _0$ . With this setting, the information will flow back into the system from the reservoir. The numerical results are presented in Fig. 5. As we predict, the *x* and *z* components of the Bloch vector track the trajectory of the instantaneous steady state $\rho _0(t)$ (see Fig. 5a), and the *y*-component of the Bloch vector does not cross the zero point (see Fig. 5b), which leads to reasonable control parameters as shown in Fig. 5c.

**Figure 6:**
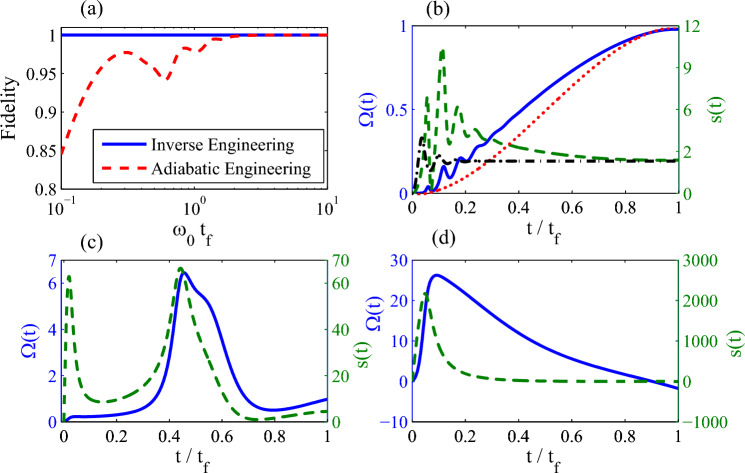
(**a**) the fidelity for the inverse steady state engineering protocol (the blue solid line) and the adiabatic engineering protocol (the red dash line) with the dimensionless parameter $\omega _0 t_f$. The control parameters as a function of the dimensionless time $t/t_f$ with different $t_f$ ((**b**) $t_f=10\omega ^{-1}$, (**c**) $t_f=\omega ^{-1}$, and (**d**) $t_f=0.1\omega ^{-1}$). In Fig. 6b, the red dot line and the black dash-dot line denote the control field $\Omega _0(t)$ and the Lamb shift $s_0(t)$ in the reference Liouvillian, respectively. Parameters: $\gamma _0=\omega _0,\,\lambda =0.2\omega _0,\,\Delta =0.5\omega _0,\,\delta =0.1\omega _0,\,\Omega _c=\omega _0.$ We set $\omega _0=1$ as the unit of the other parameters.

In Fig. 6a, we plot the fidelity between quantum states governed by the control Liouvillian $\hat{{\mathcal {L}}}_c(t)$ (blue solid line) (the reference Liouvillian $\hat{{\mathcal {L}}}_0(t)$ (red dash line)) and the quantum state $\rho _r(t)=1/2\left( I+r_x(t)\sigma _x+r_y(t)\sigma _y +r_z(t)\sigma _z\right)$ with the designed trajectory given by Eqs. (36) and (37). The quantum state engineered by the inverse engineering scheme always tracks the designed trajectory, but the final fidelity approach to 1 only in the long-time limit. Even though the quantum state arrived at the target state at the end of the adiabatic control process in the long-time limit, it still deviate the designed trajectory within the control process due to the oscillation of the deocherence strength. In other words, the adiabatic trajectory tracking can not be achieved for the non-Markovian dynamics. We also present the control parameters $\Omega (t)$ and *s*(*t*) with different control periods $t_f=10\omega _0 ^{-1}$ (Fig. 6b), $t_f=\omega _0^{-1}$ (Fig. 6c), and $t_f=0.1\omega _0^{-1}$ (Fig. 6d). As we see in those figures, more fast to drive the quantum state into the target state is, stronger control fields are required. This elucidates the trade-off between speed and energy cost, namely, that instantaneous manipulation is impossible as it requires an infinite cost.

As we have illustrated by examples about the trajectory tracking control of the non-Markovian two-level system, Eq. (33) presents a map from the trajectory of the quantum state $\rho (r_x, r_y, r_z)$ to the control parameters. By using these control parameters, the quantum system will be driven into the desired target state along an exact trajectory $(r_x, r_y, r_z)$ in the Bloch sphere. There are three issues should be mentioned: firstly, when we consider the trajectory given by the instantaneous steady state, it is not difficult to verify that ${r}^{2}+\,{r_{z}}^{2} +2\,{{r_{z}}}=0$, which leads to all of the control parameters to be meaningless (see Eq. (33)). It tells us that the adiabatic trajectory given by the instantaneous steady state $\rho _0(t)$ can not be tracked by means of the adiabatic engineering protocol. At least, one of components of the Bloch vectors has to deviate from the adiabatic trajectory, which also verifies that the adiabatic evolution for the non-Markovian dynamics does not exist. Secondly, *s*(*t*) and $\Omega (t)\,\propto r_y(t)^{-1}$, which require that $r_y$ can not be zero for this control protocol. When the dynamics is non-Markonian, $r_y$ in the adiabatic trajectory oscillates with time. Due to $r_y(t)\propto \Gamma _0(t)$, it can not be avoided that $r_y(t)$ crosses the zero point when $\Gamma _0(t)=0$. However, the inverse mixed state engineering protocol supports the selection of the trajectory of the quantum state in the Hilbert space. Therefore, we can select a trajectory whose *y*-component of the Bloch vector does not cross the zero point. Thirdly, since the differential equation Eq. (35) is about $r_x^2(t)$, the solution of Eq. (35) can not be negative. A suitable choice of $r_y(t)$, $r_z(t)$ and $t_f$ is very essential for this inverse engineering protocol. Therefore, the optional nature of the trajectory choice provides a broader prospect for the optimal control method of STAs.

## Conclusion

n this paper, we have demonstrated the effectiveness of the mixed-state inverse engineering scheme in speeding up the adiabatic steady state process of open two-level quantum systems. Using a control Liouvillian based on the same framework as the reference Liouvillian, we can drive the open quantum system into the target state along the adiabatic trajectory with perfect fidelity, although incoherent control is required. We have also shown that a pure coherent control protocol can be designed to drive the quantum state from the initial steady state to the target steady state. This approach can also be used for trajectory tracking control of non-Markovian systems by keeping the reservoir parameters invariant and finding a proper trajectory.

Our results demonstrate the potential of the mixed-state inverse engineering scheme for controlling open quantum systems with coherent control techniques, which is a future development trend in quantum control theory. Extending our ideas to infinite dimensional open quantum systems and open many-body quantum systems is another interesting line of research. Experimentally, our method can be realized in feasible models, such as cavity quantum electrodynamics systems^34^, superconducting circuits systems^35^, nitrogen-vacancy centers systems^36^, and even many-body and spin-chain models^37^. The coherent control schemes in our protocol can be adopted within a reasonable parameter regime used in experiments. If incoherent control is indispensable, reservoir engineering technology can be used to continuously vary the parameters of the environment^38,39^. Therefore, our control protocol can be tested in currently available experimental situations.

## Data Availability

Te datasets used and/or analysed during the current study available from the corresponding author on reasonable request.
